# Natural Killer Cells for Immunotherapy – Advantages of the NK-92 Cell Line over Blood NK Cells

**DOI:** 10.3389/fimmu.2016.00091

**Published:** 2016-03-14

**Authors:** Hans Klingemann, Laurent Boissel, Frances Toneguzzo

**Affiliations:** ^1^NantKwest, Inc., Culver City, CA, USA

**Keywords:** NK-92 cells, immunotherapy, cancer therapy, ADCC, cellular cytotoxicity

## Abstract

Natural killer (NK) cells are potent cytotoxic effector cells for cancer therapy and potentially for severe viral infections. However, there are technical challenges to obtain sufficient numbers of functionally active NK cells from a patient’s blood since they represent only 10% of the lymphocytes and are often dysfunctional. The alternative is to obtain cells from a healthy donor, which requires depletion of the allogeneic T cells to prevent graft-versus-host reactions. Cytotoxic cell lines have been established from patients with clonal NK-cell lymphoma. Those cells can be expanded in culture in the presence of IL-2. Except for the NK-92 cell line, though, none of the other six known NK cell lines has consistently and reproducibly shown high antitumor cytotoxicity. Only NK-92 cells can easily be genetically manipulated to recognize specific tumor antigens or to augment monoclonal antibody activity through antibody-dependent cellular cytotoxicity. NK-92 is also the only cell line product that has been infused into patients with advanced cancer with clinical benefit and minimal side effects.

The remarkable responses recently achieved with T cells expressing chimeric antigen receptors (CARs) to target tumor antigens, especially in patients with lymphoid malignancies (1–3), highlight the ability of immune cells to become powerful therapeutic agents. However, in a significant number of patients, CAR-T-cell treatment was associated with adverse events including a potentially fatal “cytokine release syndrome” requiring ICU admission. In addition, the logistics and costs of this treatment pose a significant challenge for making it available for a larger number of patients. An increasing number of investigators believe that cellular therapy with natural killer (NK) cells obtained from the peripheral blood of either the patient (autologous) or a healthy donor (allogeneic) may represent safer effector cells for targeted cancer cell therapy than T cells.

However, there are biological, logistical, and financial challenges for the application of blood NK cells as a treatment modality for cancer patients (Figure 1). Autologous NK cells are typically not very effective as they are functionally silenced when they encounter self-MHC antigens, and they are also frequently compromised by the underlying disease and its treatment. On the other hand, allogeneic NK-cell infusions carry the risk of graft-versus-host (GvH) reactions even after the CD3 lymphocytes have been depleted (4). “Supply” is also limited, in part, because only about 10% of circulating blood lymphocytes are NK cells: to collect sufficient numbers of NK cells, patients or donors often have to undergo repeated leukaphereses that at times requires placing a central venous line, which is a major inconvenience for patients. This also usually limits the number of collections of NK-cell products for treatment to one or two. Moreover, to reach therapeutically meaningful numbers, NK cells have to be expanded *ex vivo*. This is most effectively done by culturing the cells (for allogeneic cells, this is after T-cell depletion) on a genetically engineered feeder layer of K562 cells that has been modified to express stimulatory molecules, such as IL-15 or IL-21 and 4-1BB (5–7). While expansion of NK cell can be achieved, some of these protocols result in NK-cell telomere shortening and reduction in cytotoxicity. Additionally, and in contrast to T-cell therapies, the ability to target blood NK cells through a CAR type mechanism is challenging due to the low transfection efficiency of blood NK cells even when viral-based methods are used.

**Figure 1:**
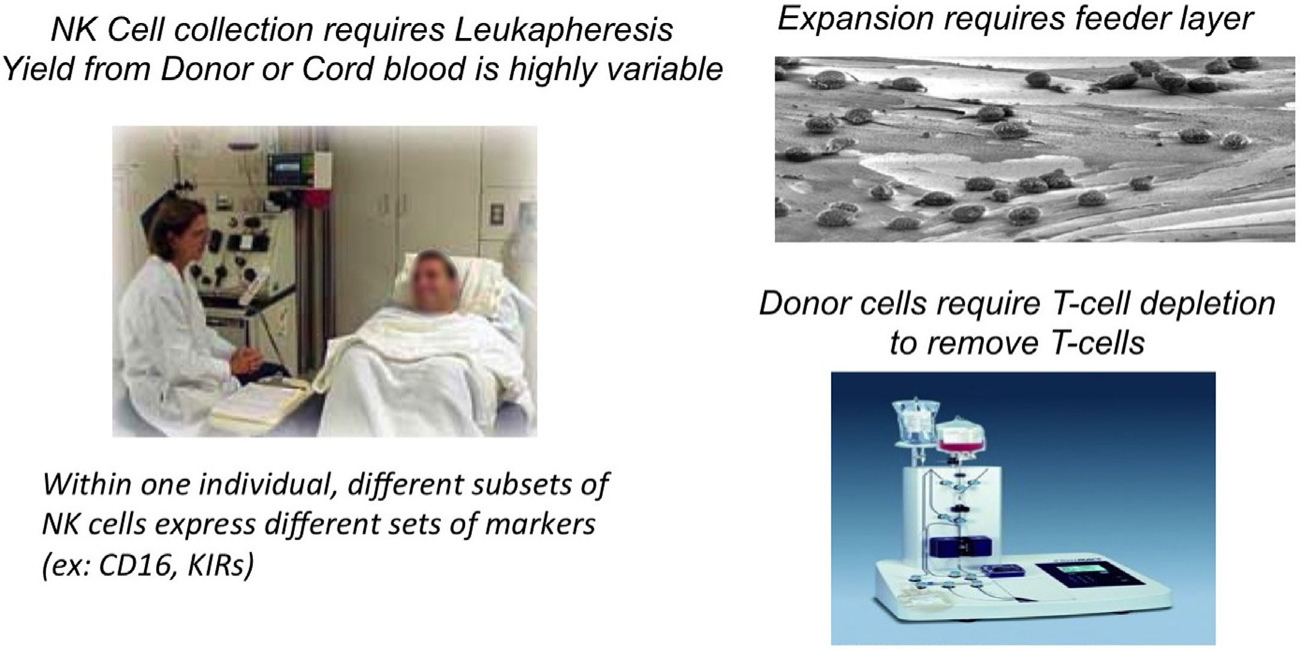
**Biologistic challenges of obtaining blood NK cells for cell therapy**.

Recognizing the significant challenges being faced in the use of blood-derived NK cells for therapeutic purposes, investigators have been trying to generate stable cell lines from blood NK cells. These efforts have generally been unsuccessful as those (frequently EBV-transformed) NK cells undergo only limited number of divisions before they experience apoptosis. The derivation of functional NK cells from embryonic stem cells and/or iPSC cells may be another avenue to generate sufficient numbers of NK cells for infusion. However, these studies are still at a relatively early stage and require additional characterization of the final product, as well as standardization of protocols, before this approach can be considered clinically relevant (8–10).

Another way of generating larger numbers of cytotoxic NK cells for treatment is *via* a clonal cell line immortalized from a patient who has developed a NK-cell lymphoma. However, NK-cell lymphoma is a relatively rare disease, and importantly, the clonal outgrowth of a cell line is a rare event. Over the past 20 years, only a handful of clonal NK-cell lines have been established (11–17) (Table 1). Those cell lines generally consist of “pure” NK cells, which proliferate and expand easily in culture, with a doubling time of 2–4 days and hence can be given to patients repeatedly on a flexible schedule. Most of those NK-cell lines do not display a robust and more universal cytotoxicity that would warrant their further development with the exception of NK-92, which is the only cell line that is consistently and highly cytotoxic to cancer targets (13). NK-92 cells have undergone extensive preclinical development (18–21) and have completed phase I trials in cancer patients [(22, 23), clinical trials NCT00900809 and NCT00990717]. Importantly, NK-92 cells – in stark contrast to blood NK cells – can be easily engineered by non-viral transfection methods to express specific receptors or ligands that can retarget them toward malignant cells.

**Table 1:** **NK-cell lines derived from patients with NK-cell leukemia/lymphoma**.

Designation	Origin	CD16	Cytokine dependence	Cytotoxicity	Reference
NK-92	LGL – lymphoma	neg	IL-2	+++	(13)
NK-YS	NK – nasal lymphoma	neg	IL-2	(+)	(16)
KHYG-1	LGL – leukemia	neg	IL-2	++	(17)
NKL	LGL – leukemia	pos	IL-2	+	(15)
NKG	LGL – lymphoma	neg	IL-2	++	(12)
SNK-6	NK – nasal lymphoma	neg	IL-2	Not tested	(14)
IMC-1	LGL – leukemia	pos	IL-2	+	(11)

Infusing cells of malignant origin may be counterintuitive, but a large body of evidence suggests that it is indeed safe as the cells are irradiated before infusion. Irradiation prevents *in vivo* proliferation while maintaining their ability to kill target cells and produce immune active cytokines. For NK-92, functional cytotoxicity is maintained after irradiation with 1000 cGy, a dose that completely abrogates proliferation (24). A large dataset in immunocompromised SCID mice has demonstrated that NK-92 cells are not tumorigenic (20, 21, 25, 26). This is supplemented with data from close to 50 patients who have now been treated with repeated infusions of irradiated NK-92 cells without any short- or long-term complications, especially tumor formation. Those phase I studies also confirmed that even with cell numbers as high as 10 billion cells/m^2^, infusions are safe with no severe unexpected side effects (22, 23). At higher doses, responses were observed even for unselected end-stage patients.

Relatively few cell lines comply with the commonly accepted definition of NK cells as summarized in Table 2. YT cells, for example, do not express CD56 but are generally considered “NK-like” because they kill the MHC negative cell line K562. On the other hand, NKL and NKG cell lines are more closely related to NK-92. In fact, the NKG cell line was established by using identical culture conditions, as described for NK-92, i.e., the combination of fetal calf and horse serum, β-mercaptoethanol, and hydrocortisone as base constituents for the medium. Both the NKG and NKL cell lines have demonstrated *in vitro* cytotoxicity against a variety of malignant target cells, but these cells have never been administered to patients (12, 15).

**Table 2:** **Operational definition of NK-cell lines**.

Parameter	Characteristics
Derivation	From NK-cell malignancy
Immortalization	+++
Monoclonality	+++
TCR genes	In germline
Morphology	Azurophilic granules, large cells
Immunophenotype	CD1^−^, CD2^+^, sCD3^−^, cyCD3ϵ^+^, CD4^−^, CD5^−^, CD7^+^, CD8^−^, CD16^−^, CD56^+^, CD57^−^, TCRαβ^−^, and TCRγδ^−^
Karyotype	Numerical and structural alterations
NK activity	+++
EBV	±

The remainder of the NK-cell lines listed in Table 1 has variable cytotoxicity toward cancer cell lines or primary malignant cells. One explanation may be that these cell lines express inhibitory KIR receptors, which are missing on NK-92 (less well characterized for NKL and NKG). For NK cells to engage and release their cytotoxic granules, adhesion molecules and the expression of activating receptors (such as NKp30, NKp44, and NKp46) are also essential. The combination of expression of activating receptors and adhesion molecules, together with the lack of most of the currently known KIRs, accounts for the broad cytotoxicity of NK-92 (27).

## Preclinical Studies in SCID Mice with NK-92

A large number of SCID mice studies with infusing either irradiated NK-92 (1000 cGy to mirror the clinical protocols) or non-irradiated NK-92 cells have been reported for a spectrum of human cancer xenotransplanted malignancies. In addition to AML (21), myeloma (28), and melanoma (20) using the parental NK-92 cells, CAR-modified NK-92 have been shown to eliminate AML [CD33.CAR (29)], lymphoma [CD19.CAR (18)], myeloma [CS1.CAR (25)], prostate cancer [EpCAM.CAR (30)], breast cancer [Her-2.CAR (31)], neuroblastoma [GD2.CAR (32)], and glioblastoma [EGFR.CAR (33)]. In those studies, CAR-modified NK-92 cells (now called taNK = targeted NK cells) eliminated the human tumor and significantly improved survival without any side effects in the recipient mice (34).

An advantage of the NK-92 platform is the ability to transfect the cells with a gene of interest without using retrovirus or lentivirus, as is necessary for T cells and peripheral blood NK cells. NK-92 can be genetically engineered by simple electroporation. Since the cells are highly IL-2 dependent, this can be used as a selection marker: the gene/construct of interest is cloned into a bicistronic vector with an IL-2 variant that is restricted to the endoplasmic reticulum and thus avoids any safety issues associated with secreted IL-2. Only those cells that are successfully transfected will grow out in a medium without IL-2, a huge advantage of a cell line over blood cells that makes the NK-92 cell platform an “off-the-shelf” engineered cellular product (35).

## Clinical Trials with NK-92

Four phase I trials in three different countries (US, Canada, and Germany) for different malignancies have been conducted with NK-92. All patients had treatment-resistant advanced cancer. The initial trials in Chicago and Frankfurt enrolled patients with renal cell and lung cancer and other solid tumors (22, 23). Two to three infusions of escalating dose levels of NK-92 were given 48 h apart. The MTD in these trials was largely based on the number of NK-92 cells that could be expanded over 2–3 weeks, and 10^10^ cells/m^2^ was considered the highest dose level. Except for some mild fever reactions in the occasional patient, the infusions were well tolerated. Despite the advanced disease, clinically significant responses were seen in patients with melanoma, lung cancer, and kidney cancer.

The study at Princess Margaret in Toronto (Keating, unpublished) enrolled patients with hematological malignancies, some of whom had relapsed after an autologous stem cell transplant. Again, those infusions were well tolerated and some clinically significant responses were noted. A phase I study at Pittsburgh Cancer Center is currently enrolling the last cohort of patients with relapsed/treatment-resistant AML. Those patients had a high leukemic blast infiltration in the bone marrow, posing a potential risk for tumor lysis syndrome, which, however, was not observed. Some patients showed a decrease or stabilization of their bone marrow blast count.

Despite the allogeneic nature of NK-92 cells and repeated infusions, the formation of HLA antibodies only occurred in less than half of all patients. This is likely related to the fact that cancer patients are immunocompromised, but it also mirrors earlier *in vitro* data suggesting that NK-92 cells are only mild stimulators in a mixed lymphocyte reaction (NantKwest, unpublished).

The costs of preparation and administration of NK-92 are significantly less compared to autologous or allogeneic NK cells and, particularly, compared to CAR.T cells, a treatment that has garnered significant attention recently. In contrast to CAR.T cell protocols, which involve highly selected patients and are believed to cost on the order of $250,000 or more, infusion cycles with engineered NK-92 cells are generally less than $20,000, with the option of repeated treatment cycles (Table 3).

**Table 3:** **Comparison between CAR-T cells and taNK**.

CAR-T	taNK
• Limited availability (T-cell “fitness”) • Donor variability • Complex collection procedure	• NK-92 is donor-independent, off-the-shelf product

• Requires precise logistics between the production facility and the treatment center	• Frozen product can be provided to treatment center as needed

• Costimulation with CD28 and/or 4-1BB	• First generation CAR sufficient

• Transfection with virus supernatant	• Electroporation of plasmid or mRNA

## The Next Generation of Engineered NK-92: haNK and taNK

**Figure 2:**
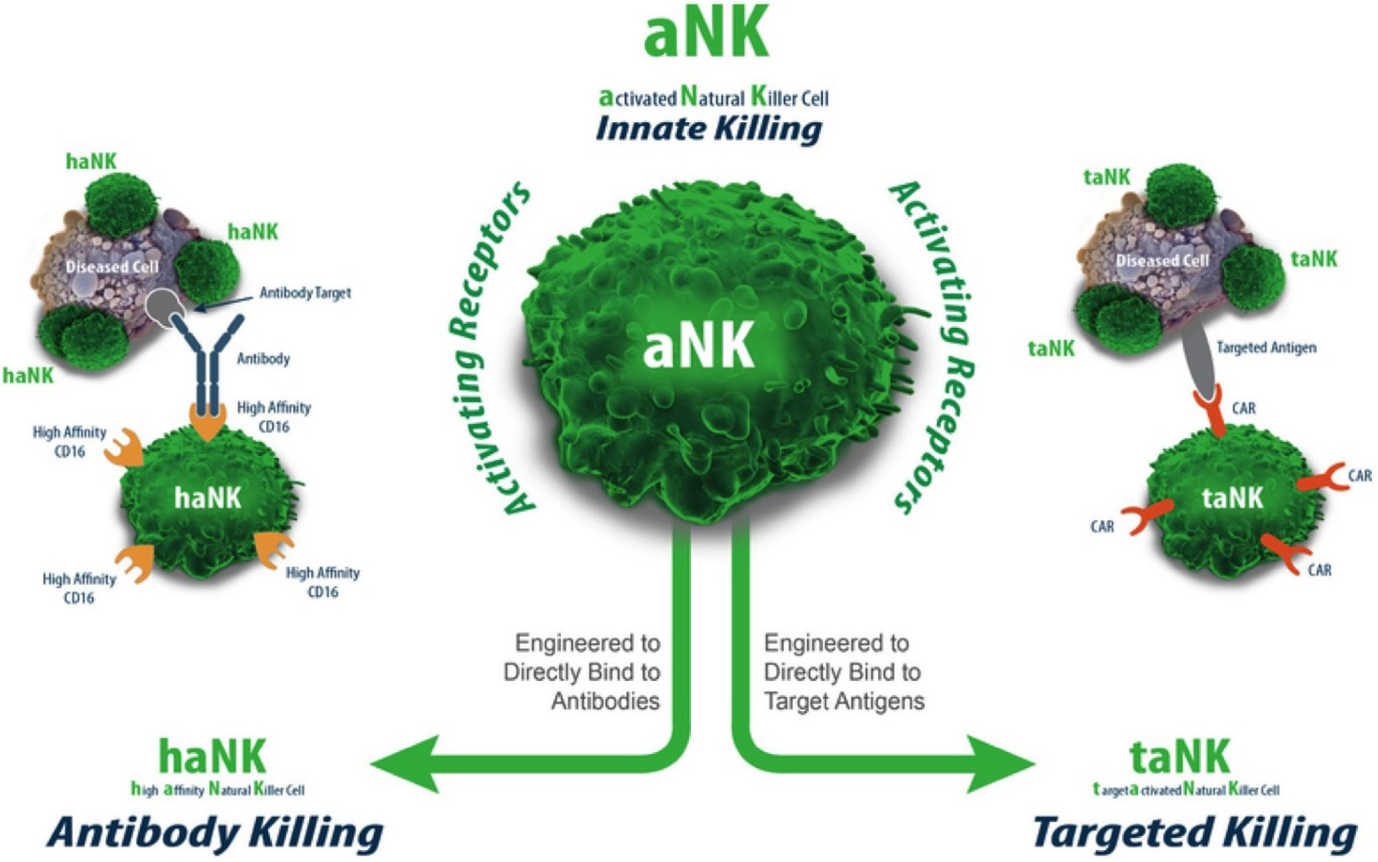
**aNK, haNK, and taNK**.

The parental NK-92 cells do not express the FcγRIIIa receptor (CD16) (Figure 2). Therefore, NK-92 cells cannot mediate antibody-dependent cellular cytotoxicity (ADCC). A NK-92 variant that expresses the high-affinity Fc receptor FcγRIIIa (158V) (haNK) is in clinical development to be combined with IgG1 monoclonal antibodies (mAbs). *In vitro* and *in vivo* studies have confirmed that combination with FcγRIIIa (V/V) augments mAb efficacy (36–39). The rationale for a treatment that combines mAb treatment with haNK infusions is based on the number of retrospective studies demonstrating an improved overall survival benefit in patients expressing the high-affinity FcγRIIIa receptor upon treatment with mAbs, such as Rituxan^®^ (lymphoma), Herceptin^®^ (breast cancer), and Erbitux^®^ (colon cancer) (36, 37, 39, 40). Since only 10% of the population is homozygous for the high-affinity FcγRIIIa receptor (V/V), there is a clear rationale for infusing haNK to those patients who carry the low- or intermediate-affinity FcγRIIIa receptor (90% of the population) (41) to maximize mAb efficacy.

The term “taNK” refers to targeted NK-92 cells (42). Those cells have been transfected with a gene that expresses a CAR for a given tumor antigen. A large body of preclinical murine data supports this approach as one with superior efficacy to the parental aNK cells [reviewed in Ref. (34)]. Further, the efficient transfection of NK-92 with mRNA (>80%) provides a route for quickly assessing the effectiveness of any given CAR construct for a particular indication (43). This approach may also ultimately provide a timely approach for personalized treatment based on a patient’s particular tumor antigen/mutation.

## The Path to Personalized Cancer Therapy

Currently, only CARs that recognize common known tumor antigens are used to transfect T cells. What is needed are CARs that recognize patient-specific tumor antigens. This “missing link” can be achieved by using proteomic analysis of patient tumors to identify patient-specific neoantigens, followed by the screening of an antibody library for that particular antigen. Gene sequencing alone is not sufficient as many somatic DNA changes in tumors do not translate into expression of tumor antigens. Based on the nucleotide sequence of the antibody’s antigen binding site, a single chain Fv (scFv) for the CAR specific for the patient’s cancer can be engineered and transfected into NK-92 cells *via* mRNA or other approaches.

**Figure 3:**
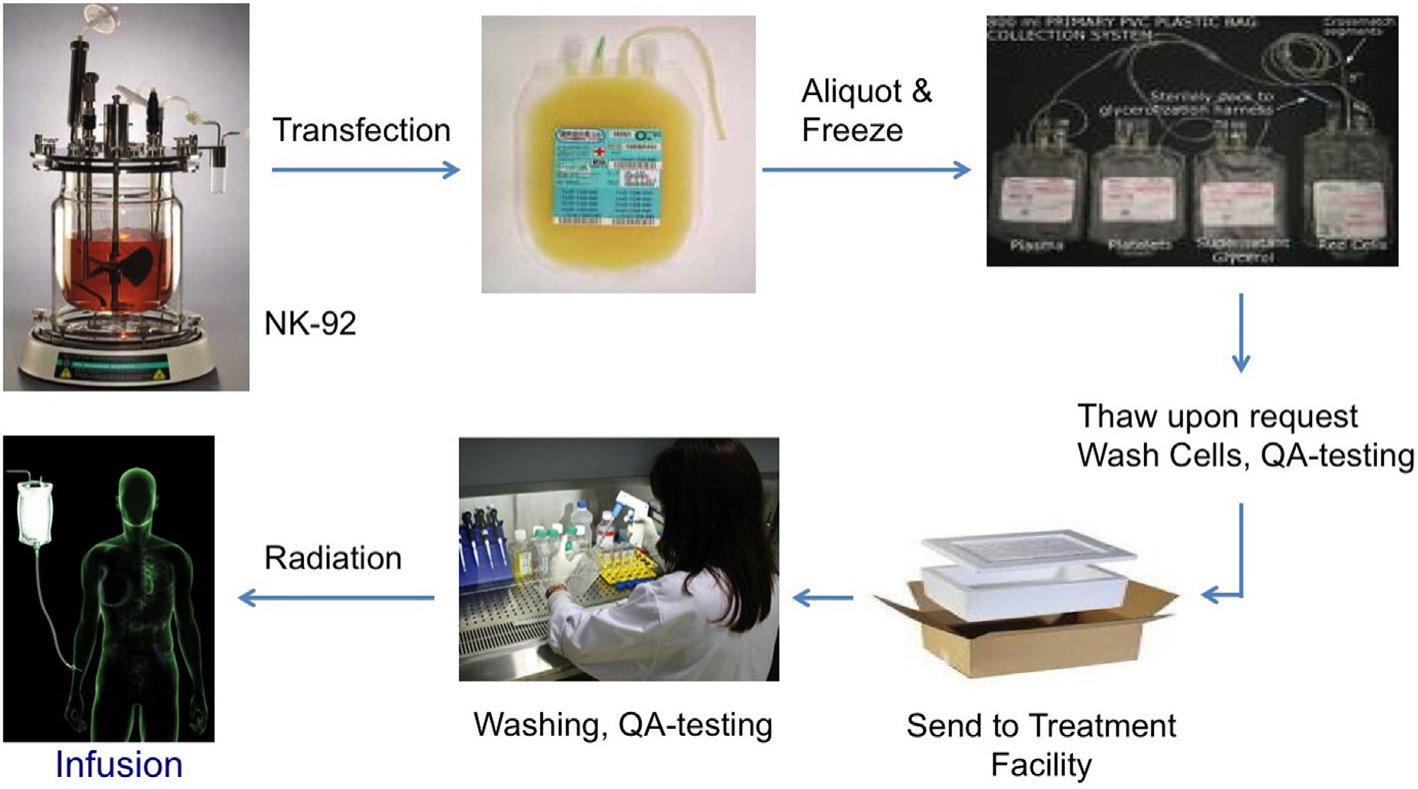
**Biologistics of NK-92 as an “off-the-shelf” cellular therapeutic**.

The non-viral, mRNA-based off-the-shelf CAR technology allows to generate large numbers of taNK that are highly specific to the patients’ tumor (“precision medicine,” Figure 3). These cells can be frozen and shipped to the treatment site, on short notice. By identifying multiple patient-specific cancer antigens, the technology also enables the engineering of alternative and overlapping CARs in the event of a change in the tumor antigen profile (“escape”).

## Homing and Target Recognition

For NK cells to get to the site of tumor, they have to express certain “homing” molecules, such as CXCR4 for bone marrow and CCR7 for lymphoid tissue (44, 45). There is also some suggestion that CXCR2 is responsible for targeting cytotoxic cells to solid tumors (46). Although the expression of a CAR probably can account for some homing, the migration of cells from the blood stream into the bone marrow, lymph nodes, or solid tumors requires appropriate trafficking and homing receptors. Once the cells are at their “destination,” the CAR will help targeting the malignant cells among the healthy ones.

## Combination Therapy

An off-the-shelf cell line, such as NK-92, with all its modifications lends itself to combination therapy. A recent review summarized the additive and synergistic effect of certain drugs (bortezomib, IMiDs, and HDAC inhibitors) on the function of blood NK cells (47) and NK-92 cells (48, 49).

The checkpoint inhibitors (Keytruda^®^, Opdivo^®^, and Yervoy^®^) have recently shown some remarkable responses in several types of cancers. This beneficial effect is believed to be largely due to blocking of inhibitory molecules on T cells, such as CTLA-4 and PD1. Studies on the expression of checkpoint molecules on activated NK cells are somewhat inconclusive, but blood NK cells seem to express PD1 (50). By using checkpoint inhibitors in combination with NK-cell therapeutics, it could be expected that both the innate and the T-cell immune response can be further augmented.

The NK-92 platform clearly provides a base for targeting tumors through a multiplicity of approaches. The platform has been proven to be safe and effective even in its unmodified (parental) form. Additional improvements through genetic modifications will provide a combination therapy approach with mAb therapy (haNK) and a direct targeting approach through CAR modification (taNK). As an off-the-shelf therapy that can be administered universally to patients, this platform can provide a cell therapy modality that is not only versatile but that can be tailored to specific patient needs.

## Author Contributions

HK contributed the main effort for writing the manuscript; LB and FT provided support for writing and review of the references, as well as for editing, formatting, and revisions.

## Conflict of Interest Statement

HK, LB, and FT are currently employed by Nantkwest, Inc.
